# Influence of Partial Acid Hydrolysis on Size, Dispersity, Monosaccharide Composition, and Conformation of Linearly-Branched Water-Soluble Polysaccharides

**DOI:** 10.3390/molecules25132982

**Published:** 2020-06-29

**Authors:** Cristina Lupo, Samy Boulos, Laura Nyström

**Affiliations:** Department of Health Science and Technology, Institute of Food, Nutrition and Health, ETH Zurich, 8092 Zurich, Switzerland; cristina.lupo@hest.ethz.ch (C.L.); samy.boulos@hest.ethz.ch (S.B.)

**Keywords:** water-soluble polysaccharides, dietary fibers, partial acid hydrolysis, fractional precipitation with isopropanol, monosaccharide ratio, Mark-Houwink-Sakurada relationship

## Abstract

The effect of partial acid hydrolysis on the physical and chemical properties of galactomannan, arabinoxylan, and xyloglucan was investigated. Polysaccharides were treated at 50 °C with hydrochloric acid for 3–48 h. Portions of isopropanol (*i*-PrOH) were added sequentially to the hydrolyzates, resulting in fractions that were collected by centrifugation. As expected, a significant reduction of weight-average molecular weight (*M_w_*) was observed with increasing hydrolysis time. Fractional precipitation was successfully applied to collect at least one polymer fraction with dispersity (*Đ*) close to one for each polysaccharide. The monosaccharide composition analysis showed that the partial hydrolysis usually lowered the relative amount of side chains, with the exception of galactomannan, where the composition remained largely unaffected. Estimation of the polymer conformation in solution, through evaluation of the Mark-Houwink parameter coefficient (*α*), confirmed that acid hydrolysis influenced the polysaccharides’ conformation. It was demonstrated that acid treatment in dilute solution followed by fractional isopropanol precipitation is a method, extendible to a variety of polysaccharides, to obtain materials of decreased molecular weight and low dispersity with slightly altered overall composition and conformation.

## 1. Introduction

Many water-soluble polysaccharide applications are based on their physicochemical behavior in aqueous solutions. For example, intrinsic viscosity is strongly correlated to the molecular weight (*M_w_*), and the polymer conformation is affected by many parameters such as the *M_w_*, dispersity of the sample, its degree of branching, and its connectivity in the backbone [1,2,3]. The dispersity (*Đ* = *M_w_*/*M_n_*) is a dimensionless number used to characterize the molecular weight distribution of polymeric materials [4]. Considering basic research, analytical purposes, food applications, encapsulation or drug delivery system applications, polysaccharides with a high homogeneity in molecular weight are often preferred to obtain a uniform high-quality product [5]. In addition, since the solubility of a polysaccharide decreases with the increase of its molecular weight, lower *M_w_* polysaccharides are often chosen, especially considering their applications in the food industry as functional foods. Hence, it is useful to develop a method to produce a collection of polysaccharides with low *M_w_* and low dispersity. A characterization of these polymers in terms of size, dispersity, chemical composition, and conformation is essential to find a correlation between their properties and their potential industrial implementation. For example, a study carried out by Mikkonen and colleagues [6] revealed that a lower degree of polymerization in galactomannan-based films significantly improved the elongation at break and tensile strength of films. Kochumalayil and Berglund [7] used xyloglucan to study its strength, stiffness, and oxygen barrier performance for polymer packaging application. They discovered that a removal of the galactose residues in the side chain by enzymatic modification improved the mechanical properties of the film under high humidity conditions.

**Figure 1:**
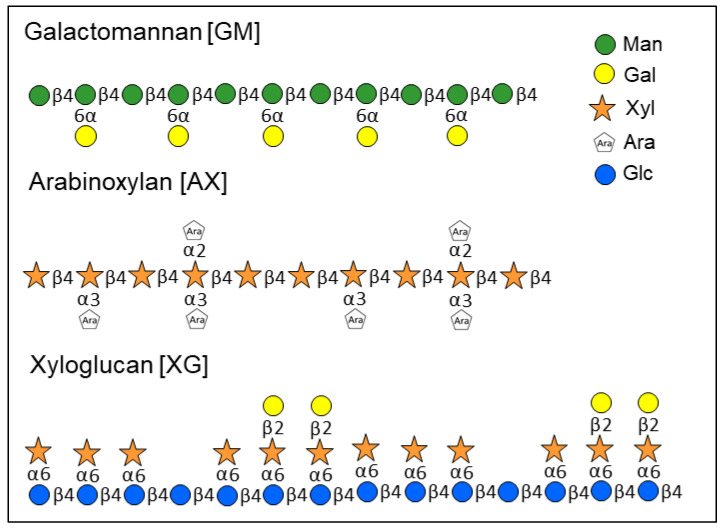
Schematic representation of the chemical structures of galactomannan, arabinoxylan, and xyloglucan structure.

Among the many existing natural polymers, particular attention has been paid to the so-called linearly-branched water-soluble polysaccharides, which consist of short branches on an essentially linear backbone. These molecules have intermediate physicochemical properties between those of perfectly linearly and fully branched polysaccharides [8]. For example, arabinoxylan, galactomannan, and xyloglucan belong to this group (see Figure 1). Galactomannan, a linear polysaccharide consisting of a backbone of β-(1→4)-linked D-mannose randomly substituted with α-(1→6)-linked galactopyranosyl units, has thickening and gelling properties, which make it an excellent food-product additive. It was also reported that galactomannan can improve the shelf-life of food products by binding water [9]. Another polymer that finds application in various fields is arabinoxylan. It is composed of a linear backbone of β-(1→4)-linked D-xylopyranosyl residues randomly substituted with α-L-arabinofuranosyl units at the O-2 and/or O-3 position. Isolated arabinoxylan is mainly applied as cryostabilizer for food and drug preparations, in film formation for packaging production, and for coating food products in order to improve quality, safety, and shelf-life [10,11]. Researchers have also shown a particular interest in xyloglucan, which is made of a β-(1→4)-linked D-glucan backbone partially substituted at the O-6 position with α-xylopyranosyl branches that could be further derivatized by β-(1→2)-linked galactopyranosyl residues. Xyloglucan is often used as fat replacement and gelling agent [12]. 

Although the native compounds of these linearly-branched polymers already find numerous applications in industrial fields, the product resulting from their depolymerization owns physical features that make the hydrolyzed material more suitable for many other applications. For example, hydrolyzed guar gum is used as a low-viscosity fracturing fluid in hydraulic fracturing applications for oil and gas well stimulation [13]. Hydrolyzed xyloglucan (average degree of polymerization (DP) = 17) was employed as an additive in bread-making preparation. The stability of the dough, the loaf volume, and the softness of the bread was considerably improved after the addition of depolymerized xyloglucan [14]. Finally, oral administration of partially hydrolyzed arabinoxylan (*M_w_* = 53 kDa), was successfully applied in mice to increase their immunopotentiating activity reaction without provoking an immunological reaction [15].

To achieve the desired physicochemical properties, polysaccharides can be subjected to depolymerization through several procedures, such as microwave or ultrasound irradiation, or enzymatic and chemical cleavage. Using irradiations (gamma or X-rays) for polymer degradation enhances yields and reduces the reaction time. However, irradiation may lead to radical formation, which can further react and change the primary structure of polysaccharides [16,17,18]. Sonication exploits the energy that is released when bubbles, produced during a high-pressure cycle sound-waves transmission, collapse [19]. Although sonication may improve the reaction rate of the depolymerization without significantly changing the chemical composition and dispersity of the starting materials, and is considered a green process technique, a variety of operating parameters must be considered in advance. Besides the ultrasonic intensity and frequency, other factors such as starting molecular weight, concentration of the polymer solution, and even the operating reaction volume can affect the extent of the degradation process [20]. Enzymatic cleavage, on the other hand, is a regio- and stereoselective reaction leading to high yields and a minimum of unfavorable chemical modifications. Despite that, the performance of enzymatic hydrolysis is strictly correlated with the ability of the enzyme to tailor a particular substrate under specific buffer, pH, and temperature conditions [21]. Of the chemical cleavages, a controlled acid hydrolysis is a relatively easy, inexpensive, and universal method for polysaccharide depolymerization. However, it is known that acid hydrolysis is less specific than an enzymatic approach as all the glycosidic bonds are susceptible to breakage to varying degrees, potentially leading to increased heterogeneity [22]. 

Fractional precipitation is a procedure that consists of a gradient addition of, for example, alcohol to a polysaccharide solution, mainly to isolate and fractionate polysaccharides molecules based on their different molecular weight and solubilities. Although a large amount of data has been reported on the precipitation behavior of different sources of polysaccharides either with ethanol or isopropanol [23], the fractionation of partially hydrolyzed polysaccharides as a method to reduce dispersity is still unexplored. 

In this study, we created a method for acid catalyzed depolymerization and fractionation of water-soluble polysaccharides that led to samples with lower *M_w_* and dispersity. Guar galactomannan, wheat arabinoxylan, and tamarind xyloglucan were chosen as polysaccharide substrates, belonging to the soluble dietary fibers, a food compound class with established beneficial health effects such as blood cholesterol lowering. They have a positive effect on inflammatory bowel diseases and they have an important role in preventing obesity [24,25]. The different monosaccharide composition both in the backbone polymer chain structure and type and degree of substitution make them interesting substrates to compare their behavior under the action of acid hydrolysis followed by precipitation with isopropanol. These polysaccharides are therefore a smart choice, both for their physical properties and for their health beneficial value, when it comes to their integration into functional foods. Moreover, their specific properties such as solubility, water-holding capacity, viscosity, and gelling properties as well as binding ability and fermentability are the key factors that make these polysaccharides useful for other applications such as processing aids in food production, drug delivery systems, and pharmaceutical/cosmetic preparations.

Galactomannan, arabinoxylan, and xyloglucan were hydrolyzed by hydrochloric acid in dilute solutions. The products were fractionated with isopropanol to obtain fractions with lower dispersity. Native and treated samples were characterized in terms of molecular weight distribution, monosaccharide ratio, and chain conformation. In addition, the hydrolysis kinetics of *M_w_* as a function of time and type of polysaccharide was investigated. This study contributes to a better characterization of the physicochemical properties of three water-soluble polysaccharides, and demonstrates a universal and affordable method for the realization of a library composed of various low molecular weight water-soluble polysaccharides with reduced dispersity, which can be potentially used in a number of industrial applications.

## 2. Results and Discussion

### 2.1. Chain Scission Kinetics of Acid Hydrolysis and Dispersity Analysis 

The chain scission kinetics of the partial hydrolysis process for all polysaccharides was investigated by the following equation:

(1) $$\frac{m_{0}}{M_{n(t)}}=k\cdot t+\frac{m_{0}}{M_{n(0)}}$$

**Figure 2:**
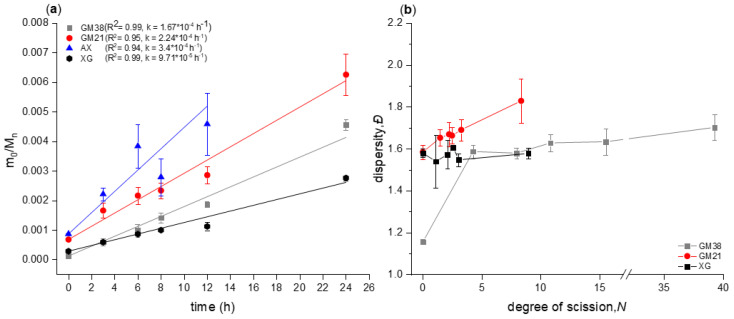
(**a**) Degradation kinetics of the chain backbone of polysaccharides during acid hydrolysis. Galactomannan (GM38 and GM21) and arabinoxylan (AX), 0.1 M HCl at 50 °C. Xyloglucan (XG), 0.4 M HCl at 50 °C. (**b**): Dispersity (*Đ* = *M_w_*/*M_n_*) as function of the degree of scission *N*, namely the average number of glycosidic bonds cleaved per polymer molecule (AX data not shown due to analytical artefacts (see text)).

Whereas m_0_ is the average repeating unit molar mass, and *M_n(t)_* and *M_n_*_(0)_ are the number-average molecular weights [g/mol] at time *t* and time 0, respectively. The term on the left-hand side of the equation indicates the number of broken bonds per anhydro monosaccharide unit. By plotting the inverse of the degree of polymerization (1/*DP_n_* = m_0_/*M_n_*) over the time course of the degradation, the rate at which a polysaccharide chain breaks during a partial acid hydrolysis was obtained. This plot with m_0_/*M_n_* = 1/*DP_n_* representing the increase in relative amount of reducing ends was done for guar galactomannan (samples GM38 and GM21 with different degrees of galactose substitution), arabinoxylan (AX), and xyloglucan (XG) (see Figure 2a). A linear relationship between m_0_/*M_n_* and hydrolysis time was established for GM38 (R^2^ = 0.99), which is in agreement with Equation (1), confirming that glycosidic linkages within the polysaccharide backbone were equally susceptible to cleavage, and hence random chain scission occurred with a rate constant (*k*) of 1.67·10^−4^ h^−1^. An almost perfect positive linear relationship between m_0_/*M_n_* and time of hydrolysis was found (Pearson’s correlation coefficient (r) = 0.99), indicating that the number of broken bonds per anhydro-monosaccharide unit was strictly related to the hydrolysis time. A similar trend was observed for the hydrolysis of GM21. The coefficient of determination (R^2^) was 0.95, Pearson’s correlation coefficient (r) was 0.97, and the rate constant (*k*) was 2.24·10^−4^ h^−1^ indicating that the reaction was faster for GM21 than GM38 as obvious from the steeper slope (Figure 2a). A lower degree of galactose substitution on the mannose backbone in GM21 seems to positively influences the rate of cleavage. XG followed a similar trend as GM38 denoting that the chain scission followed a random cleavage. The rate constant (*k*) was 9.71·10^−5^ h^−1^, the coefficient of determination (R^2^) was 0.99 and, Pearson’s correlation coefficient (r) was 0.99. Arabinoxylan had the highest kinetic rate constant (*k*) of 3.4·10^−4^ h^−1^ confirming that glycosidic linkages between aldopentopyranoside rings are hydrolyzed faster than linkages between aldohexopyranoside rings. 

Although most of the studies related to the mechanism of action involved in the hydrolysis of polysaccharides are yet to be understood, some hypotheses can be formulated by comparing the different kinetic rate constants (*k*) obtained for the hydrolysis of galactomannan (GM38, GM21), arabinoxylan (AX), and xyloglucan (XG).

Up to now, the most accepted mechanism that explains the process by which a polysaccharide is hydrolyzed is the “cyclic” type. Briefly, the glycosidic oxygen atom is protonated by the acid, rendering it a good leaving group under the formation of an oxocarbenium ion, which exists in the half-chair conformation and which in turn reacts with a water molecule to form the reduced sugar [26]. It is also known that the glycosidic bond at the non-reducing end is hydrolyzed faster than an internal bond. The formation of oxocarbenium ion is accompanied by a change of the sugar unit conformation, for which C-2, C-1, O, and C-5 are in a planar conformation. Therefore, the hydrolysis of an internal bond would involve a reorientation of the bulky group

Timell reported the rate constants and kinetic parameters for eight disaccharides undergoing acidic hydrolysis with 0.5 M sulphuric acid [27]. Of interest for our study are the values found for cellobiose, mannobiose, xylobiose that can be compared with our chain scission rate constant obtained for xyloglucan, galactomannan, and arabinoxylan. Considering the β-(1→4)-linked disaccharides, it was found that the relative rates of hydrolysis were in the order of xylose > galactose > mannose > glucose. This is in agreement with the trend shown in the Figure 2, where the rate of chain scission was in the order of arabinoxylan > galactomannan > xyloglucan. It was demonstrated by BeMiller that a restriction in the flexibility of the molecules in the ground state and in the transition state, measure by the entropy of activation (ΔS^‡^), decreases the rate of the hydrolysis [28]. For linearly-branched polysaccharides the degree of substitution in the backbone chain can influence the freedom of motion of the molecules in the ground state and in the transition state. This could explain why galactomannan with higher degree of substitution (GM38) hydrolyzed slower than the one with less degree of substitution (GM21).

The variation of the dispersity index (*Đ*) as a function of degree of scission (*N*) was investigated for all the studied polysaccharides. The degree of scission

(2) $$N=\frac{M_{n(t=0)}}{M_{n(t)}}-1$$

indicates the fraction of broken bonds per polymer chain during acid hydrolysis (see Figure 2b). GM38 increased its dispersity value from 1.15 ± 0.01 to 1.70 ± 0.06. A similar result was found for GM21, its dispersity increased significantly from 1.58 ± 0.03 to 1.83 ± 0.12 (*p* = 0.0109). For XG, the dispersity remained constant as degradation proceeded with an approximate value of 1.6, indicating that large chains were degraded with the same probability as smaller chains. All these results are largely consistent with the theoretical prediction for a random-chain scission model according to the following function [29]

(3) $$\underset{N\to \infty }{\lim}Đ=2,$$

where *N* is the degree of scission and *Đ* is the dispersity (*M_w_*/*M_n_*). As reported by Yoon et al. [29], three conditions may occur when a random chain scission happens. When *Đ* of the native polymer is less than 2, it will increase up to 2 during the degradation. If it is greater than 2, it will decrease to 2, and if the initial value is close to 2, the dispersity will remain constant during the hydrolysis process. 

**Figure 3:**
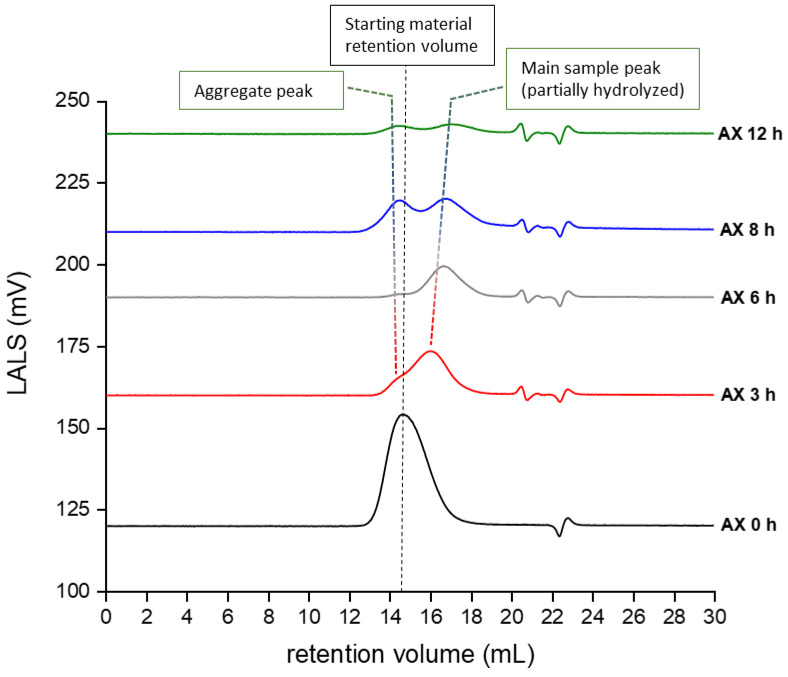
Low-Angle Light Scattering (LALS) ignal of native (AX 0 h) and partially hydrolyzed AX (AX 3–12 h). After 3 h of treatment with HCl, a second peak was observed in the LALS signal, which becomes more predominant as the hydrolysis time increases. This peak corresponds to aggregates formed during the partial hydrolysis reaction that are able to significantly scatter the laser light. The system used for the analysis could not sufficiently separate the hydrolyzed peak from the aggregates peak; therefore, the integration of the peak related to the hydrolyzed sample was impeded, making the dispersity measurement not reliable.

Concerning AX, it was not possible to determine the appropriate values of m_0_/*M_n_* and *Đ* by integrating the polymer peak in the chromatogram, due to the formation of aggregates after 3 h of hydrolysis (see Figure 3).

As expected, partial hydrolysis followed the random chain scission model, and the time of hydrolysis seems to be the main factor influencing the relative amount of reducing end per polymer chain produced during the process. Moreover, the degree of substitution on the backbone polymer chain has an inverse linear correlation with the reaction rate constant, as shown by comparing *k* of GM21, GM38, and XG. 

#### 2.1.1. Conformational Analysis

All the samples were also analyzed for their conformational properties in solution using the Mark-Houwink-Sakurada equation, which describes the relationship between the intrinsic viscosity ([*η*]) and *M_w_*:

(4) $$\begin{array}{c} \left[\eta \right]={K\cdot M_{w}}^{\alpha }\\ \log\;([\eta ])=\log\;(K)+\alpha \cdot \log\;(M_{w}) \end{array}$$

**Figure 4:**
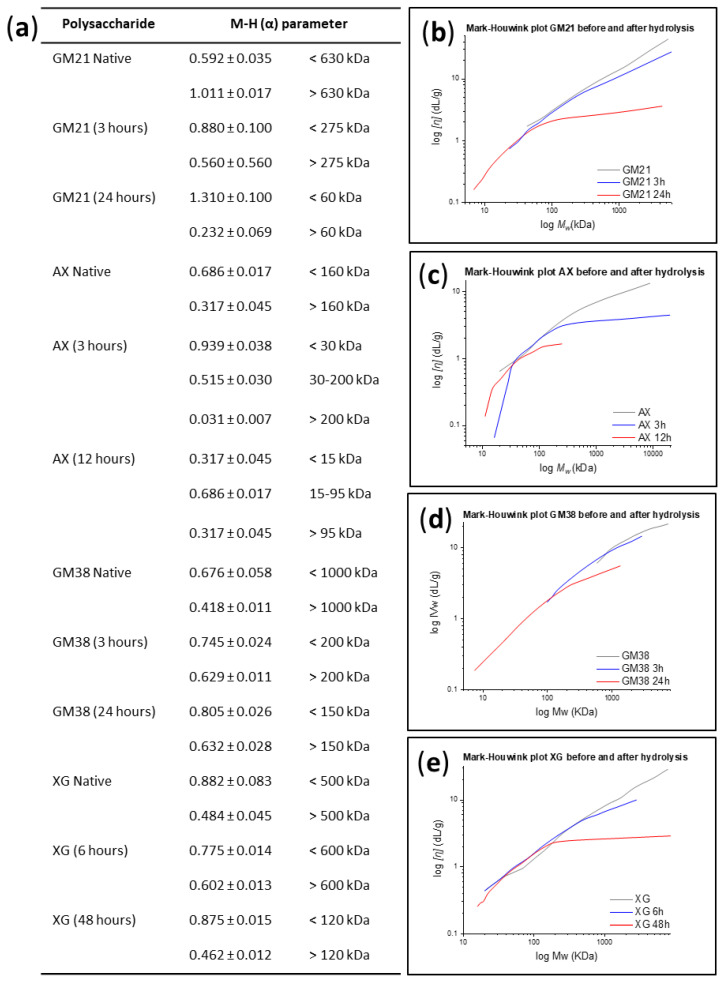
(**a**) Mark-Houwink α values of polysaccharides before and after partial hydrolysis. Mark-Houwink-Sakurada plots, namely the variation in intrinsic viscosity ([η]) as a function of weight average *M_w_* of (**b**) guar galactomannan (GM21), (**c**) wheat arabinoxylan (AX), (**d**) guar galactomannan (GM38), and (**e**) tamarind xyloglucan (XG) before and after partial acid hydrolysis (3, 12, or 24 h). The data were acquired by SEC-RID-LALS/RALS-IV using 0.1% polysaccharide in aqueous 0.1 M NaNO_3_ + 0.02% NaN_3_ solution.

Using a double log plot of the intrinsic viscosity [*η*] versus *M_w_* (Equation (4)), it was possible to determine the average conformation of the polymer in solution for each polymer sample (*α*) [30]. From the Mark-Houwink plot analysis, it was shown that the curves of all the native and hydrolyzed polysaccharide samples had two or more deflections along the molecular weight distribution, and therefore, more than one α value was determined (see Figure 4a–d). For example, guar galactomannan (Gal:Man = 21:79) treated with HCl for 24 h (GM21 (24 h)) consist of compact polymer chains up to a *M_w_* ~ 60 kDa (*α* = 0.232 ± 0.069), and more stiff-rod like chains (*α* = 1.310 ± 0.140) when the molecular weight is higher than 60 kDa. Considering arabinoxylan hydrolyzed for 3 h with HCl (AX (3 h)), it was possible to define even three regions in the curve with different *α* values. A stiff conformation was observed for *M_w_* < 30 kDa, a more extended for 30 kDa < *M_w_* < 200 kDa and, a contracted conformation for *M_w_* > 200 kDa. Except for native XG at lower molecular weight (*M_w_* < 500 kDa) and native GM21 at higher molecular weight (*M_w_* > 630 kDa), for all other native polysaccharides, *α* values ranged between 0.5 and 0.7, with Mark-Houwink plot not deviating much from a linear pattern throughout the whole molecular weight distribution. This *α*-range suggests that native AX (at *M_w_* < 160 kDa), GM38 (at *M_w_* < 1000 kDa), GM21 (at *M_w_* < 630 kDa), and, XG (at *M_w_* > 500 kDa) adopted the same overall random coil conformation in solution. However, from the native to the hydrolyzed samples, the *α* value changed, meaning that the hydrolysis was, to some extent, influencing the hydrodynamic property of the polysaccharides. In particular, newly produced low molecular weight fractions adopted a stiff rod-like chain conformation.

#### 2.1.2. Relationship Between Hydrodynamic Radius and Intrinsic Viscosity

The correlation between intrinsic viscosity [*η*] and hydrodynamic radius (*R_h_*) of native and hydrolyzed polysaccharides, at a given concentration and solvent, was investigated (see Table 1). Intrinsic viscosity and hydrodynamic radius are sensitive to the extension of a polymer chain and their interrelation can be used to hypothesize the supramolecular organization of a macromolecule in solution. For all the polysaccharides, [*η*] and *R_h_* decrease with increasing hydrolysis time in accordance with their decreasing *M_w_* (see Table 2, Table 3, Table 4 and Table 5). Lower values of [*η*] and *R_h_* indicate a more condensed structure and higher molecular density. The resulting compact structure is in agreement with the α values reported in Figure 3a (e.g., α _(GM21 Native)_) = 0.592 ± 0.035 (*M_w_* < 630 kDa); 1.011 ± 0.017 (*M_w_* > 630 kDa) and α _(GM21 (24h)_ = 1.305 ± 0.1040 (*M_w_* < 60 kDa); 0.232 ± 0.009 (*M_w_* > 60 kDa)) which indicated that acid treatment led to a stiffer conformation.

Moreover, the intrinsic viscosity is independent from the molecular weight of a polysaccharide species, in the sense that two polysaccharide structures with comparable M_w_ may have different [*η*]. For example, the intrinsic viscosity of GM21_(Native)_ (M_w_ = 380 kDa, [*η*] = 6.61 ± 0.04 g/L) was almost twice the number of AX_(Native)_ (M_w_ = 320 kDa, [*η*] = 3.57 ± 0.04 g/L). Therefore, the highest intrinsic viscosity for GM21_(Native)_ implies a more open structure (higher R_h_) and lower density. Another parameter that increases the density in solution is the degree of branching. This is in agreement with the intrinsic viscosity values of GM21 and GM38. The lower degree of galactose substitution on GM21 backbone made its intrinsic viscosity in solution lower than that of GM38. The effect of partial acid hydrolysis on the viscoelastic properties of galactomannan, arabinoxylan and xyloglucan under dynamic shear condition, as a measure of the elastic and viscous material response, could be a valuable future study for exploring structure and performance of the newly produced hydrolyzed fractions.

#### 2.1.3. Molecular Weight and Dispersity Analysis of Hydrolyzed and Fractionated Polysaccharides 

*M_w_* and *Đ* of all native, hydrolyzed, and precipitated polysaccharide fractions were analyzed using HPSEC. Guar polymer chains GM21 and GM38 (see Table 2 and Table 3) were significantly degraded to a lower *M_w_* after 3 and 24 h of hydrolysis, while *Đ* increased over time.

Changes observed in molecular properties of XG during 6 and 48 h of hydrolysis (see Table 4) were comparable to those observed with GM21 and GM38 (see Table 2 and Table 3).

A comparable *M_w_* reduction was found for arabinoxylan after 3 and 12 h of hydrolysis; however, Đ decreased during depolymerization (see Table 5).

Comparing the two galactomannans after 3 and 24 h of hydrolysis, we found that the quantity of isopropanol necessary to start the precipitation was higher for GM38 (16 and 19% after 3 and 24 h hydrolysis, respectively) than for GM21 (14 and 15% after 3 and 24 h hydrolysis, respectively). Most likely, the higher degree of galactose substitution on the mannose backbone discourages the formation of intermolecular interaction, requiring more alcohol to initialize the precipitation for GM38. In addition, considering GM21 after 24 h hydrolysis, its stiffness (*α* = 1.305, *M_w_* < 60 kDa), might help the aggregation process. Intermolecular interaction between polymer segments are privileged for a stiff rod conformation since polymer chains can simply assemble together, leading a precipitate formation. Instead, for a random coil conformation (0.5 < *α* < 0.8), polymer chains need first to rearrange nicely to optimized the intermolecular interaction [31]. 

Gradient precipitation is based on the solubility of the polymer, which in turn depends on its *M_w_*. The more soluble a polysaccharide is, the higher alcohol concentration is needed to initiate the precipitation. Polysaccharide fractions usually precipitate in a descending order of molecular weight with increasing isopropanol concentration. 

**Figure 5:**
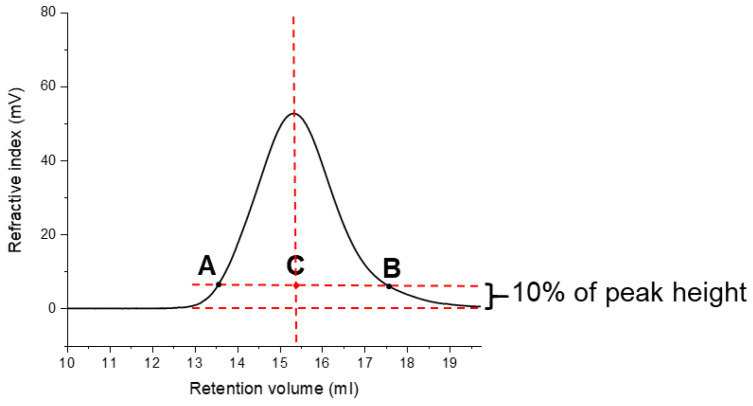
The asymmetry factor (As) measures the peak tailing and is defined as the distance from the center line of the peak to the back slope divided by the distance from the center line of the peak to the front slope, measured at 10% of the maximum peak height.

**Figure 6:**
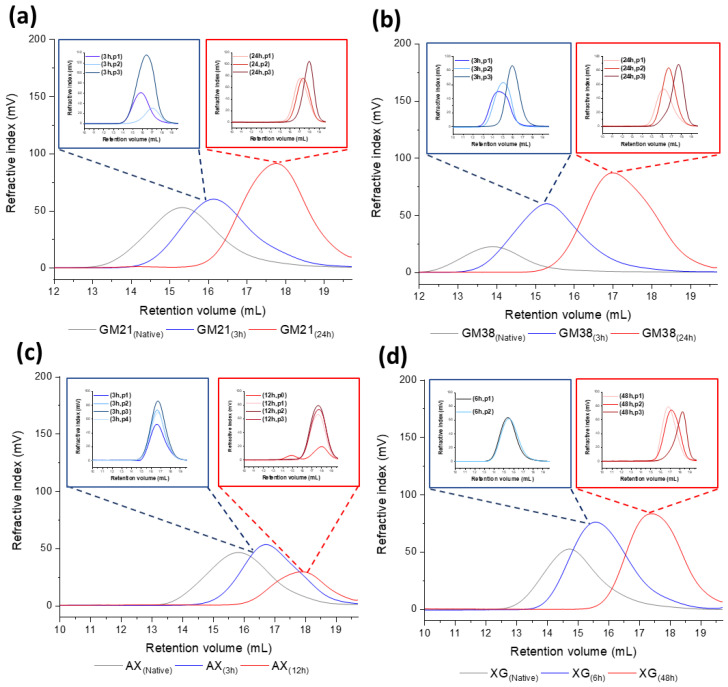
MWD plots measured by high- performance size exclusion chromatography (HPSEC) (OMNISEC) of native and partially hydrolyzed polysaccharides. (**a**) GM21_(Native)_, GM21_(3h)_, and GM21_(24h)_; (**b**) GM38_(Native)_, GM38_(3h)_, and GM38_(24h)_; (**c**) AX_(Native)_, AX_(3h)_, and AX_(12h)_; (**d**) XG_(Native)_, XG_(6h)_, and XG_(48h)_. The inserts (blue and red boxes) shows the MWD plots of the isolated fractions of hydrolyzed samples after precipitation with *i*-PrOH.

Four and three fractions of arabinoxylan were obtained with *i*-prOH precipitation after 3 and 12 h of hydrolysis, respectively (see Table 5). For both AX (3 h) and AX (12 h), the substitution degree positively correlates with isopropanol concentration, which is in agreement with Dervilly, Saulnier [32]. With the increase of Ara residues as side chains, the polysaccharides become more water soluble, demanding more isopropanol to accomplish the precipitation. However, considering AX (3 h), its consecutive fractions were collected in an ascending order of molecular weight, not following the rule that the highest molecular weight molecules would precipitate first (see Table 5). The backbone weight average molecular weight ($M_{w}^{AX\left(\mathrm{pi},backbone\right)}$ = $M_{w}^{AX\left(pi\right)}$ (% Xyl)) of AX (3 h) fractions, such as p1 with p3, were very comparable ($M_{w}^{AX\left(p1\right)}$ = 84 kDa, $M_{w}^{AX\left(p1,backbone\right)}$ = 61 kDa; $M_{w}^{AX\left(p3\right)}$ = 114 kDa, $M_{w}^{AX\left(p3,backbone\right)}$ = 80 kDa). Therefore, for AX (3 h) the gradient precipitation depends mainly on the solubility of the polysaccharide fractions controlled by the substitution pattern than their *M_w_*. For GM21, no significant difference was found on the average molecular weight of the polysaccharide fractions, and for AX, only the *M_w_* of first fraction p1 was significantly different from the hydrolyzed sample. Therefore, their *M_w_* are not apparently related to isopropanol concentration. In addition to the dispersity parameter, the asymmetry factor (A_s_) (see Figure 5) and the molecular-weight distribution functions (MWDs) of native, hydrolyzed, and precipitated polysaccharide fractions were investigated (see Figure 6). Particular attention was given to the symmetry of the function with respect to the maximum point of the Gaussian distribution (number-average molecular weight), which is related to the absolute composition of the polymer chain lengths [33]. From almost all of the distribution functions shown in Figure 6, we observed that the curves did not deviate significantly from a normal distribution function, meaning that the samples consist of the same statistical segment length. However, GM21_(24h)_, GM38_(24h)_, GM38_(24h,p3)_, and XG_(24h,p3)_ deviated from the symmetrical shape. The asymmetry factor (A_s_) is a measure of the polysaccharide’s skewness and it is defined as:

(5) $$A_{s}=\frac{\mathrm{BC}}{\mathrm{CA}}$$

When a distribution is negative skew (As < 1), the polymer population contains a larger fraction of low molecular-weight polymer chains, contrariwise when the shape is positively skew (As > 1) a larger fraction of high molecular-weight chains is present.

For GM21_(24h)_, the distribution shifted to the higher region of molecular weight (As = 0.9), while for GM38_(24h)_ was the opposite (As = 1.4). GM38_(24h,p3)_ and XG_(24hp3)_ both showed a negative skew trace.

In conclusion, with the exception of AX (3 h), for all the other precipitated samples, we obtained fractions with dispersity lower than the original hydrolyzed samples before fractionation. The best results with regard to low dispersity were achieved for the second precipitate of GM21 and GM38 hydrolyzed for 24 h and 3 h, respectively (${Đ}_{GM21}^{24h,p2}\;$ = 1.004 ± 0.002, ${Đ}_{GM38}^{3h,p2}$ = 1.193 ± 0.003). Partial acid hydrolysis significantly degraded the *M_w_* of the native polysaccharides, and isopropanol precipitation was a valid method for obtaining polymer fractions with lower dispersity than the hydrolyzed polysaccharide fractions. Finally, with few exceptions, acid hydrolysis coupled with *i*-PrOH precipitation can control the MWD shape, leading to samples with similar chain length.

### 2.2. Monosaccharide Ratios

The monosaccharide ratio of native and HCl-treated and fractionated polysaccharide was determined using high-performance anion-exchange chromatography-pulsed amperometric detection (HPAEC-PAD) after complete hydrolysis to understand whether the partial acidic hydrolysis could affect the uniformity of the polysaccharide samples. For GM21 (3 h) and GM21 (24 h), a statistically significant difference was found between the monosaccharide ratio of the first precipitate and that of the other two precipitated fractions (see Table 2). However, two out of three fractions did not differ statistically from the native compound, emphasizing that the acid hydrolysis did not significantly affect the uniformity of the final products. No variation on the monosaccharide ratio was found when comparing the GM38 fractions within each time point with each other (see Table 3). Nevertheless, the monosaccharide ratio of the hydrolyzed fractions differed from the native polysaccharide’s ratio, indicating that the homogeneity of the polysaccharide population remains constant regardless of hydrolysis time. A strong difference in the monosaccharide ratio was observed for AX, both within a group of fractionated samples and between the fractions and the starting polysaccharide (see Table 5). Therefore, the acid hydrolysis had a significant impact in increasing the heterogeneity of the final product, or at least revealed it. The same was observed for XG (see Table 4). After 6 h of hydrolysis, the monosaccharide ratio of the collected fractions did not differ from the native xyloglucan. However, after 48 h, the percentage of galactose and xylose sidechains changed. Specifically, galactose reduced its relative content by roughly a quarter and xylose increased it by around a quarter (XG Native: Xyl:Gal = 23:15; XG_(p1)_: Xyl:Gal = 29:11; XG_(p2)_: Xyl:Gal = 27:12; XG_(p3)_: Xyl:Gal = 31:13).

Lastly, partial acid hydrolysis in combination with precipitated fractionation seems to modify the uniformity of the studied polysaccharides, or at least reveal their heterogeneity. In particular, the highly substituted galactomannan (GM38), arabinoxylan, and the longest hydrolyzed xyloglucan (XG 48 h) seem to increase their heterogeneity after partial hydrolysis.

## 3. Materials and Methods

### 3.1. Materials

The properties of the native polysaccharides provided from Megazyme (Bray, Ireland) were as follows: Guar galactomannan high viscosity (Lot#100301 a, viscosity > 10 dL/g, *M_w_* = 380 kDa and sugar ratio Gal:Man = 38:62 (GM38). Guar galactomannan high viscosity, Gal depleted (Lot#10502 a), viscosity > 10 dL/g, *M_w_* = 350 kDa and sugar ratio Gal:Man = 21:79 (GM21). Wheat flour arabinoxylan medium viscosity (Lot#40601), viscosity 31.4 cSt, *M_w_* = 323 kDa and sugar ratio Arabinose:Xylose = 38:62 (AX). Tamarind xyloglucan (Lot#150901), viscosity 14 dL/g, *M_w_* = 800 kDa and sugar ration Xylose:Glucose:Galactose:Arabinose:Other sugar = 31:49:17:2:1 (XG). All polysaccharides were used without any further purification. The *M_w_* provided in the product specifications by Megazyme were confirmed by size exclusion chromatography with the exception of GM38, which was more than four times larger than the reported *M_w_*. This specific *M_w_* was already reported in other studies [34,35]. Concentrated hydrochloric acid (HCl, 37%), sodium phosphate dibasic (Na_2_HPO_4_, 99.95%), isopropanol (*i*-PrOH, ≥ 99.7%), sodium hydroxide solution (NaOH, 50–52% in water), and D-sorbitol (extra pure for microbiology) were purchased form Sigma-Aldrich (St. Louis, MO, United States). All aqueous solutions were prepared using MilliQ-water (Millipore). Dialysis membranes made from regenerated cellulose with MWCO 12′000-14′000 Da (25 Å; 29 mm) were supplied by SERVA (Heidelberg, Germany).

### 3.2. Acid Hydrolysis in Dilute Solution

Partially hydrolyzed polysaccharides were prepared in triplicates based on a method by Whistler and Durso [36] with some modifications. Solutions of 0.6% (*w*/*v*) polysaccharide were prepared in water by hydrating the weighed polysaccharide for 1 h at 80 °C under stirring (800 rpm). Samples were cooled to room temperature under mechanical stirring over night to ensure a complete dissolution of the polymers. Partial hydrolysis was initiated by adding concentrated HCl to reach 0.1-M HCl final concentration for galactomannan and arabinoxylan, whereas 0.4 M HCl was used in the xyloglucan solution. The reaction times were selected based on pre-experiments to achieve two polysaccharide samples with a statistically significant different molecular weight, and the experiment was conducted at 50 °C for 3 and 24 h for galactomannan, 3 and 12 h for arabinoxylan, and 6 and 48 h for xyloglucan. To quench the reaction and neutralize the polysaccharide solutions (6 ≤ pH ≤ 7), equal volumes of 0.2 M Na_2_HPO_4_ or 0.4 M Na_2_HPO_4_ were added to galactomannan/arabinoxylan and xyloglucan, respectively. The resulting solutions were dialyzed against water for 72 h at room temperature, exchanging the water every 24 h.

### 3.3. Collection of Low Dispersity Polysaccharide Fractions by Isopropanol Precipitation

**Figure 7:**
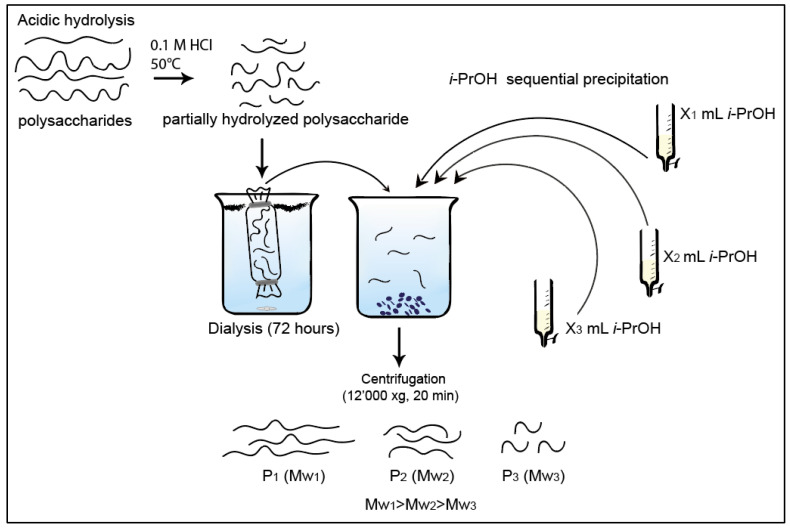
Scheme of partial hydrolysis and fractional precipitation of guar, wheat, and tamarind polysaccharides.

Dialyzed polysaccharide solutions were fractionated using isopropanol (*i*-PrOH) (Figure 7). First, volume X_1_ of *i*-PrOH was added to the polysaccharide solution until a precipitate was observed. The sample was stored on ice for 2 h and centrifuged at 12,000 g for 20 min to collect the precipitate. After this, the procedure was repeated with the remaining supernatant twice with *i*-PrOH volumes X_2_ and X_3_. All precipitates were freeze-dried for 24 h. Samples were stored in a desiccator at room temperature until further analysis.

### 3.4. Chain scission Kinetics

The chain scission kinetics of the partial hydrolysis process for all polysaccharides was investigated by the following equation:

(6) $$\frac{m_{0}}{M_{n(t)}}=k\cdot t+\frac{m_{0}}{M_{n(0)}}$$

where *M_n(t)_* and *M_n(0)_* are the number-average molecular weight [g/mol] at time *t* and time 0, respectively. The term on the left-hand side of the equation indicates the number of broken bonds per anhydro monosaccharide unit. The parameter k [h^−1^] is the rate constant for the main chain scission and m_0_ [g/mol] is the molecular weight of the repeating unit which was 162 g/mol for GM38, GM21, and XG, and 132 g/mol for AX [37]. The *M_n_* was determined by size exclusion chromatography after partial hydrolysis using the same reaction conditions as described in 3.2. Aliquots were collected after 0, 3, 6, 8, 12, and 24 h, neutralized with a solution of Na_2_HPO_4_, diluted with water up to a polysaccharide concentration of 1 mg/mL, filtered through 0.45-µm Nylon filters and analyzed by size exclusion chromatography. Previous studies already established that the chain scission mechanism of different polysaccharides follows a random process (Equation (6)) where all the linkages at any site along the chain have the same probability to be broken [38]. 

### 3.5. Molecular Weight and Dispersity Analysis

**Figure 8:**
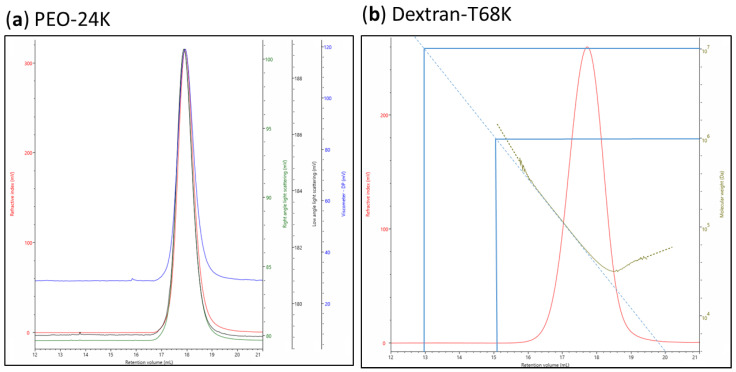
MWD plot measured by high-performance size exclusion chromatography (HPSEC) (OMNISEC) of standards: (**a**) PEO-24K for light scattering/triple detection calibration, (**b**) Dextran-T68K for validation of the calibration, with excellent agreement *M_w_* vs. retention time (mL) relation (*M_w_* (Dextran-T68K)_Malvern_ = 68162 ± 450 Da, *M_w_* (Dextran-T68K)_Validation_ = 66807 ± 291 Da).

Weight-average *M_w_* and *Đ* of each native and hydrolyzed polymer fraction were determined by high-performance size exclusion chromatography (HPSEC) (OMNISEC, Malvern Panalytical Ltd., Malvern, United Kingdom). The system consisted of an OMNISEC RESOLVE chromatography compartment combined with a pump, an autosampler, and a column oven equipped with two A’6000M columns in series (8.0 × 300 mm, Viscotek, parent organization: Malvern Panalytical Ltd., Malvern, United Kingdom). OMNISEC RESOLVE detector compartment was equipped with a low and right-angle laser light scattering detector (LALS/RALS), a refractive index (RI), a UV detector and a viscometer. OMNISEC software version v.10.30 was used for data acquisition, analysis, and reporting. A solution of 0.1 M NaNO_3_ with 0.02% NaN_3_ was used as mobile phase. The temperature of both columns was kept at 30 °C, and the flow rate was 0.7 mL/min with an injection volume of 100 µL. Samples were dissolved in the mobile phase at a concentration of 0.1% (*w*/*v*) and filtered through a 0.45-µm nylon filter prior to injection. For the absolute molecular weight determination, a calibration was performed using narrow molecular weight distribution polyethyleneoxide (PEO-24K) standard (see Figure 8) using the refractive index increment (dn/dc) value of 0.144 mL/g for galactomannan, 0.132 mL/g for arabinoxylan, and 0.146 mL/g for xyloglucan. These refractive index increments were determined using HPSEC by creating a calibration curve (RID area vs. concentration) with standard solutions of polysaccharides of various concentration (0.1–1.0%). All samples were measured in triplicates. 

#### 3.5.1. Mark-Houwink-Sakurada Relationship

The Mark-Houwink-Sakurada relationship (Equation (4)) was used to evaluate the stiffness of the backbone of the polymer chain before and after partial hydrolysis. All the samples were also analyzed for their conformational properties in solution using the Mark-Houwink-Sakurada equation, which describes the relationship between the intrinsic viscosity ([*η*]) and *M_w_*. The specific parameters *K* and α, which are related to the intercept and the slope of the plot, respectively, were obtained by plotting the *M_W_* against the intrinsic viscosity [*η*] on a double logarithmic graph [30]. 

An increase of the slope indicates a stiffening of the molecule, while a decrease indicates a more compact dense structure. Generally, when *α* = 1.8 a rigid rod conformation is expected. However, when 0.5 < *α* < 0.8, a semi-flexible or random coil conformation is usually assumed. Alternatively, if the polymer adopts a compact conformation, because it is highly branched or because of intramolecular segment-segment interactions, *α* acquires values close to 0. Intrinsic viscosity and *M_W_* values were obtained from the OMNISEC software.

#### 3.5.2. Relationship Between Intrinsic Viscosity and Hydrodynamic Radius

Intrinsic viscosity [*η*] and the hydrodynamic radius *R_h_* were given as output of the OMNISEC software. In dilute solution, the intrinsic viscosity is directly correlated to the volume occupied by the single molecule and this correlation can be expressed by the following viscosity relationship [39]:

(7) $$R_{h}={\left(\frac{3[\eta ]M_{w}}{10\pi N_{A}}\right)}^{1/3}$$

where *N_A_* is Avogadro’s constant. Higher values of [*η*] and *R_h_* at fixed *M_w_* indicate a more open structure and lower molecular density. However, a chemical modification may vary the chemical fine structure of a polysaccharide, changing its hydrodynamic properties. 

### 3.6. Monosaccharide Composition by HPAEC-PAD

High-performance anion-exchange chromatography-pulsed amperometric detection (HPAEC-PAD) (Thermo Scientific Scientific AG, Basel, Switzerland) was used to measure the monosaccharide ratio before and after the partial hydrolysis. An amount of 50 mg of dried samples were completely hydrolyzed in 10 mL 2 M HCl solution at 100 °C for 45 min. After cooling to room temperature, the reaction mixture was neutralized with 4 M NaOH and centrifuged for 15 min at 4000 rpm. The hydrolysates were diluted with water to reach a concentration of 10 mg/L and filtered through a 0.45-µm PTFE filter.

The analysis was performed with the ICS-5000 system, which included a DIONEX AS-AP autosampler module, a DIONEX IC-5000 DC compartment equipped with an electrochemical detection cell with a disposable gold electrode, an Ag/AgCl reference electrode, a CarboPAC PA1 (4 × 250 mm) column, and DIONEX ICS-5000 SP pump compartment. The temperature of the column was kept at 26 °C, and the flow rate was set at 1.0 mL/min. The mobile phase consisted of two eluents: (A) 200 mM NaOH (prepared from 50% (*w*/*w*) NaOH solution) and (B) water. An isocratic method was applied for the sugar separation. Then, 8 (A) and 92% (B) was run for the first 22.5 min followed by 100% (A) for 8.5 min and 8 (A) and 92% (B) for 8 min. The total run time was 39 min. For the determination of the absolute monosaccharide amount, an external standard calibration was performed using a mixture of standard sugars containing galactose and mannose for galactomannan samples, arabinose, and xylose for arabinoxylan samples, and glucose, galactose, xylose, and arabinose for xyloglucan samples. The standard mixture concentration ranged between 1.25–30.0 mg/L for galactomannan and arabinoxylan and 0.3–20.0 mg/L for xyloglucan. D-Sorbitol was used as internal standard and added at a constant concentration of 10 mg/L to each sample and calibrant solution. The monosaccharides concentration was quantified relative to the internal standard signal. Data processing was carried out on Chromeleon 7 (Thermo Fischer Scientific AG, Basel, Switzerland). All samples were measured in triplicate.

### 3.7. Statistical Analysis

Significant differences in average molecular weight of native, hydrolyzed, and fractionated polysaccharides as well as the monosaccharide ratio, were assessed by analysis of variance (One-way ANOVA) using ORIGIN Pro 2019 (OriginLab Corporation, Northampton, MA, USA). Value of *p*-value less than 0.05 (*p* < 0.05) indicated a significant difference. A linear fit was performed to describe the relationship of Equation (3) using ORIGIN Pro 2019. 

## 4. Conclusions

Based on the experimental results, we can conclude that the combination of acid treatment of dietary fibers and consecutive precipitation with isopropanol produces a collection of water-soluble polysaccharide fractions with a wide range of lower *M_w_* and *Đ*, leading to products with functional diversity. The most promising material was GM21, for which a polymer fraction with a dispersity close to one was obtained for *M_w_* = 7.39 kDa, and this with a reasonable, practically useful isolated yield. The other three fractions with *Đ* ≤ 1.5 were successfully collected (GM21_(3h,p1)_, GM21_(3h,p3)_, GM21_(24h,p3)_). These polysaccharide fractions can be new potential candidates for drug delivery or analytical standard applications. For all the treated polysaccharides, the hydrolysis time influenced the rate at which the polymers hydrolyzed, following a random chain scission process. In addition, species with lower degrees of substitution hydrolyzed faster than highly substituted polysaccharides. An extensive characterization of native and fractionated samples in terms of composition and hydrodynamic properties was performed by HPAEC-PAD and HPSEC. As a result of the monosaccharide composition analysis it can be concluded that partial hydrolysis may increase the heterogeneity of the polysaccharide population. The molecular features of hydrolyzed fractions were assessed by the Mark-Houwink-Sakurada relationship, confirming that partial hydrolysis influenced the hydrodynamic property of GM38, GM21, AX, and XG. Specifically, hydrolyzed samples assumed a stiff-rod like conformation at lower molecular weights. 

In conclusion, partial hydrolysis coupled with isopropanol precipitation of linearly-branched polysaccharides can be considered a universal and efficient method for the production of versatile polymers, which can be tested for various applications, to achieve a uniform high-quality product increasing their commercial value. Other linearly-branched polysaccharides may undergo the same treatment in order to create a library of polysaccharides with lower dispersity. Moreover, further research may be conducted to analyze their influence in food, pharmaceutical/cosmetic preparations, drug, and agrochemical controlled-release delivery system applications in order to broaden their use.

## Figures and Tables

**Table 1:** Intrinsic viscosity and hydrodynamic radius evolution of GM21, GM38, AX, and XG before and after partial acidic hydrolysis.

Polysaccharides	Hydrolysis Time (h)	*R_h_* (nm)	[*η*] (dL/g)
GM21	0 (native)	32.69 ± 0.15	6.61 ± 0.04
	3	19.80 ± 1.13	3.62 ± 0.31
	24	8.20 ± 0.57	0.98 ± 0.08
GM38	0 (native)	67.80 ± 1.10	12.31 ± 0.25
	3	31.90 ± 3.70	5.18 ± 0.75
	24	10.34 ± 0.98	1.25 ± 0.19
AX	0 (native)	24.57 ± 0.23	3.57 ± 0.04
	3	14.62 ± 0.82	1.95 ± 0.15
	12	7.94 ± 0.48	0.84 ± 0.05
XG	0 (native)	41.91 ± 0.35	5.69 ± 0.05
	6	24.83 ± 0.46	3.47 ± 0.09
	48	8.20 ± 0.25	0.85 ± 0.03

**Table 2:** *M_w_* and *Đ* variation of GM21 during 3 and 24 h acid hydrolysis before and after *i*-PrOH precipitation, with p1, p2, and p3 being the polysaccharide fractions collected during stepwise addition of *i*-PrOH ^#^.

Guar Galactomannan [GM21] HCl Hydrolysis						
	(%) *i*-PrOH	(%) Purity	(%) Yield	Gal:Man	*M_w_* (kDa)	*Đ* (*M_w_*/*M_n_*)
Native	-		-	19:81 a	380.00 ± 9.90 a	1.58 ± 0.03
3 h					151.00 ± 17.00 b	1.65 ± 0.02
p1	14	62	23	21:79 b	30.49 ± 0.52 c	1.03 ± 0.003
p2	18	69	31	18:82 a	13.26 ± 0.54 c	1.12 ± 0.03
p3	23	62	1	18:82 a	24.51 ± 0.36 c	1.02 ± 0.001
24 h					32.30 ± 1.40 B	1.86 ± 0.04
p1	15	73	15	16:84 c	12.44 ± 0.22 C	1.31 ± 0.02
p2	21	73	29	19:81 a	7.39 ± 0.02 C	1.00 ± 0.002
p3	26	65	2	19:81 a	4.47 ± 0.03 C	1.05 ± 0.005

(^#^) *i*-PrOH (%) (*v*/*v*) added during the fractionation of hydrolyzed galactomannan. Purity (%) of polysaccharide fractions and yield (%) determined gravimetrically of the dried polysaccharide fractions. Gal/Man, monosaccharide ratios of precipitated isolated fractions. Letters denote statistically different groups (*p*-value < 0.05, *n* = 3).

**Table 3:** *M_w_* and *Đ* variation of GM38 during 3 and 24 h acid hydrolysis before and after *i*-PrOH precipitation, with p1, p2, and p3 being the polysaccharide fractions collected during stepwise addition of *i*-PrOH ^#^.

Guar Galactomannan [GM38] HCl Hydrolysis						
	(%) *i*-PrOH	(%) Purity	(%) Yield	Gal:Man	*M_w_* (kDa)	*Đ* (*M_w_*/*M_n_*)
Native	-	74	-	41:59 a	1657.00 ± 43.00 a	1.55 ± 0.01
3 h					552.00 ± 5.00 b	1.53 ± 0.01
p1	16	92	11	37:63 b	673.00 ± 8.00 c	1.36 ± 0.02
p2	19	95	54	37:63 b	587.00 ± 4.00 b	1.19 ± 0.003
p3	23	96	5	37:63 b	178.00 ± 1.00 d	1.26 ± 0.01
24 h					76.08 ± 2.25 B	1.65 ± 0.03
p1	19	89	9	37:63 b	158.61 ± 0.33 C	1.56 ± 0.06
p2	24	91	42	37:63 b	89.98 ± 0.52 B	1.33 ± 0.005
p3	29	85	14	38:62 b	43.60 ± 0.65 B	1.44 ± 0.02

(^#^) % *i*-PrOH added during the fractionation of hydrolyzed galactomannan. % Purity of polysaccharide fractions calculated by High-Performance Anion-Exchange Chromatography (HPAEC) and % yield determined gravimetrically weighing dried polysaccharide fraction after precipitation. Gal/Man, monosaccharide ratio of precipitated fractions. Letters denote statistically different groups (*p*-value < 0.05, *n* = 3).

**Table 4:** *M_w_* and *Đ* variation of XG during 3 and 48 h acid hydrolysis before and after *i*-PrOH precipitation, with p1, p2 and p3 being the polysaccharide fractions collected during stepwise addition of *i*-PrOH ^#^.

Tamarind Xyloglucan [XG] HCl hydrolysis						
	(%) *i*-PrOH	(%) Purity	(%) Yield	Ara:Gal:Xyl:Glc	*M_w_* (kDa)	*Đ* (*M_w_*/*M_n_*)
Native	-	95	-	2:15:23:60 a	916.00 ± 10.00 a	1.57 ± 0.03
6 h					279.00 ± 17.00 b	1.66 ± 0.14
p1	15	86	30	<LOD:15:24:60 a	380.00 ± 25.00 c	1.28 ± 0.06
p2	19	64	33	<LOD:15:24:60 a	337.00 ± 2.00 d	1.40 ± 0.006
48 h					73.41 ± 0.83 A	1.66 ± 0.11
p1	17	94	17	<LOD:11:29:59 a	90.00 ± 7.10 A,B	1.38 ± 0.11
p2	25	95	48	<LOD:12:27:61 a	60.44 ± 0.46 A,C	1.28 ± 0.002
p3	27	92	2	<LOD:13:31:55 b	28.75 ± 0.42 C,D	1.33 ± 0.001

(^#^) % *i*-PrOH added during the fractionation of hydrolyzed Xyloglucan. % Purity of polysaccharide fractions calculated by HPAEC and % yield determined gravimetrically weighing dried polysaccharide fraction after precipitation. Ara/Gal/Xyl/Glc monosaccharide ratio of precipitated fractions. Letters denote statistically different groups (*p*-value < 0.05, *n* = 3).

**Table 5:** *M_w_* and *Đ* variation of AX during 3 and 12 h acid hydrolysis before and after *i*-PrOH precipitation, with p1, p2, and p3 being the polysaccharide fractions collected during stepwise addition of *i*-PrOH ^#^.

Wheat Arabinoxylan [AX] HCl Hydrolysis						
	(%) *i*-PrOH	(%)Purity	(%) Yield	Ara:Xyl	*M_w_* (kDa)	*Đ* (*M_w_*/*M_n_*)
Native	-	83	-	40:60 a	320.00 ± 3.00 a	2.08 ± 0.04
3 h					109.00 ± 7.00 b	1.91 ± 0.18
p1	18	58	6	27:73 b	84.00 ± 7.20 c	1.35 ± 0.04
p2	22	69	6	30:70 b	102.00 ± 8.00 b,c	1.40 ± 0.03
p3	27	68	5	30:70 c	114.00 ± 13.00 b,d	1.48 ± 0.05
p4	30	64	3	30:70 c	114.00 ± 8.00 b,d	1.53 ± 0.05
12 h					53.89 ± 11.30 B	1.47 ± 0.05
p0 ^Ϯ^	0	84	16	13:87 d	2360.00 ± 200.00 ^Ϯ^ D	10.56 ± 0.62 ^Ϯ^
p1	15	80	1	20:80 d	136.00 ± 22.00 C	1.49 ± 0.08
p2	21	76	20	24:76 e	53.10 ± 8.90 B	1.34 ± 0.04
p3	26	70	2	30:70 c	39.10 ± 1.70 B	1.32 ± 0.02

(^#^) *i*-PrOH (%) added during the fractionation of hydrolyzed arabinoxylan. Purity (%) of polysaccharide fractions and yield (%) determined gravimetrically of the dried polysaccharide fractions. Ara/Xyl monosaccharide ratios of precipitated isolated fractions. Letters denote statistically different groups (*p*-value < 0.05, *n* = 3). (^Ϯ^) After dialysis, a precipitate (p0) was formed and removed by ultracentrifugation prior to isopropanol addition. The *Mw* and *Đ* of this fraction p0 are a result of a considerable amount of aggregates, which were visible on the LALS/RALS and RI OMNISEC detectors.
